# A Comprehensive Review on Medicinal Plants as Antimicrobial Therapeutics: Potential Avenues of Biocompatible Drug Discovery

**DOI:** 10.3390/metabo9110258

**Published:** 2019-11-01

**Authors:** Uttpal Anand, Nadia Jacobo-Herrera, Ammar Altemimi, Naoufal Lakhssassi

**Affiliations:** 1 Department of Molecular and Cellular Engineering (MCE), Jacob Institute of Biotechnology and Bioengineering (JIBB), Sam Higginbottom University of Agriculture, Technology and Sciences, Prayagraj (Allahabad), Uttar Pradesh 211007, India; 2 Unidad de Bioquímica, Instituto Nacional de Ciencias Médicas y Nutrición Salvador Zubirán. Av. Vasco de Quiroga 15. Col. Belisario Domínguez Sección XVI. C.P. Tlalpan, Ciudad de México 14080, Mexico; nadia.jacobo@gmail.com; 3 Department of Food Science, College of Agriculture, University of Basrah, Basrah 61004, Iraq; 4 Department of Plant, Soil and Agricultural Systems, Southern Illinois University, Carbondale, IL 62901, USA; naoufal.lakhssassi@siu.edu

**Keywords:** antibiotics, infectious disease, antimicrobial resistant, secondary metabolites, immune response

## Abstract

The war on multidrug resistance (MDR) has resulted in the greatest loss to the world’s economy. Antibiotics, the bedrock, and wonder drug of the 20th century have played a central role in treating infectious diseases. However, the inappropriate, irregular, and irrational uses of antibiotics have resulted in the emergence of antimicrobial resistance. This has resulted in an increased interest in medicinal plants since 30–50% of current pharmaceuticals and nutraceuticals are plant-derived. The question we address in this review is whether plants, which produce a rich diversity of secondary metabolites, may provide novel antibiotics to tackle MDR microbes and novel chemosensitizers to reclaim currently used antibiotics that have been rendered ineffective by the MDR microbes. Plants synthesize secondary metabolites and phytochemicals and have great potential to act as therapeutics. The main focus of this mini-review is to highlight the potential benefits of plant derived multiple compounds and the importance of phytochemicals for the development of biocompatible therapeutics. In addition, this review focuses on the diverse effects and efficacy of herbal compounds in controlling the development of MDR in microbes and hopes to inspire research into unexplored plants with a view to identify novel antibiotics for global health benefits.

## 1. Introduction

Multidrug resistance (MDR) is a major cause of human suffering that impairs the trust relationships between doctors and their patients, concomitant with huge economic losses. In this world of microbe-man cohabitation, survival of the human species will be threatened without health-giving microbes, and there will be no way to survive the emergence of MDR superbugs. Hence, from a health perspective, antibiotics have been our best strategy. The impact of antimicrobial resistance (AMR) is a huge concern, which results in the greatest loss to individual and social economy [1]. It is estimated that by 2050, the death rate due to AMR will balloon to 10 million lives per year at an expense of one hundred trillion dollars [1,2]. Today the rapid development of MDR in microorganisms is increasing global health problems and presents a challenge for the treatment of infectious diseases that scientists claim could return to the level of the pre-antibiotic era [3]. Plants have played a unique holistic role for the provision of food, drugs, clothing, shelter, etc. Natural compounds have been extensively explored for new drug discoveries [4]. Indeed, plants have been used as medicines for more than 5000 years [3], as a source of antibiotics, antineoplastic, analgesics, cardioprotective, among others [5]. In the recent past, humans have been using natural compounds to ward off infections [6]. About 70–90% of the population in developing countries continue to use ancient medicines based on plant extracts [7]. The most powerful and promising elements of plants are their secondary metabolites, on which humans depend upon [8]. Significantly, natural products and their derivatives contribute to more than half of the Food and Drug Administration (FDA) approved drugs [9]. In the last two decades, most of the efforts have been attempts to discover novel therapeutics to combat MDR, especially with plants and deep-sea flora [10,11]. In general, natural products covers several interactions including the relationship between matter and life. Biological theories related to molecular biology and genetics, physiological and pathological theories, food, diseases, poisons, and antidotes. Such interactions give a wide range of possible uses for the secondary metabolites and their synthetic or semisynthetic derivates [12].

The disciplines of ethnobotany and ethnopharmacology define “medicinal plant” as those species used in traditional medicine that contain beneficial elements in healing diseases in humans and/or animals. The objective of ethnopharmacology is to develop a drug to treat patients, and ultimately to validate traditional use of medicinal plants.

Throughout human history, the isolation and identification of biologically active compounds and molecules from nature has led to the discovery of new therapeutics, prompting the improvement of the health and pharmaceutical sectors [13]. Phytochemicals revolve around the research and development (R&D) sector of the pharmaceutical industries as a source of new molecules leading to the development of new novel drugs [6]. For instance, in the oncology sector, plants have contributed more than 60% of the anti-cancer drugs, directly or indirectly. Natural products provide about 50% of modern drugs [14]. In the last three decades, antimicrobial resistance has led to the emergence of MDR, as a consequence of the repeated and regular use of single drugs for the same therapeutic target. Therefore, it is a warning for the pharmaceutical scientists to provide a new weapon against MDR and an opportunity to search for a new spectrum of antibiotics to fight AMR [15]. Natural products are a potential supply for novel biologically active compounds that could lead to the innovation of new therapeutics [16,17].

Two of the main living traditions that exist even today are the Traditional Indian Medicine (TIM) i.e., Ayurveda, and the Traditional Chinese Medicine (TCM), which have been knit together to contribute to diverse knowledge of therapeutic plants. Both of them have potentially contributed with a long list of plants and phytomedicines used nowadays around the world, and their input to regulate herbal drugs in the pharmacy industry [18]. In China, TCM is already playing an important role in treating infectious diseases [19] and several potential compounds are already undergoing clinical trials from Ayurveda [20]. In both traditional systems (TIM and TCM), medicines were prepared as herbal products in different formulations such us powders, tinctures, poultices, and teas to be used based on the type of disease being treated. Herbal medicines are a special branch of traditional knowledge about life dealing with both body and mind [21]. A vast majority of the global population depend on the traditional medicines for health care. Concerns are being raised related to the development of MDR as well as the side effects caused due to the introduction of drug molecules. The synthesis of Salvarsan, an arsenic based drug for syphilis in 1910, the development of Prontosil, a sulpha drug in 1935, and a penicillin purified and produced in early 1940s led to the opening of the door for future drug discovery research. The wide spread antibiotic resistance, which is observed currently, is causing public health concerns by medical researchers warning about a return to the pre-antibiotic era [22]. To date, there are no effective antimicrobial, which could cure all bacterial infections. Knowledge about the traditional medicine is undergoing generational loss of this wisdom [23]. The wide range of AMR mechanisms used by the pathogens includes:

- Enzymatic inactivation
- Modification of drug targets
- Mechanical protection provided by biofilm formation.

## 2. New Approaches for Herbal Drugs Usage: *in silico* Drug Discovery

Plants produce diverse compounds (secondary metabolites) during their lifetime, a consequence of the metabolic activities occurring within them. During the last decade, most of the ethnopharmacology resources were available in the form of comprehensive medical manuals. One of them is the Ayurvedic Pharmacopoeia of India [24]. These manuals do not provide suitable methodologies for in silico screening application. Researchers have developed several databases that store all the information that will accelerate the discovery of novel herbal drugs. Undoubtedly, database technologies opened the door for new and multiple avenues to facilitate access to higher levels of data complexity (Table 1). There are various libraries constructed by computational chemists for natural products that aim to provide excellent resources for screening and the selection of natural products [25]. Fortunately, different databases have been developed with the aim of in silico screening of ethnopharmacology records [26,27]. The diverse range of phytochemicals is being exploited by chemoinformatics [28], which ultimately accelerates the pipeline of drug development [29]. Several other databases for drug development can be explored from this link (https://www.drugbank.ca/databases). In silico High-throughput Screening (HTS), based on molecular docking, is frequently used to minimize the drugs for in vitro and in vivo screening. Thus, HTS is a powerful tool for screening the maximum number of natural compounds in a very short time to identify potential drugs.

The most important paradigm shift was the recent discovery of the powerful antimalarial drug, Artemisinin, which is derived from *Artemisia annua* L. (sweet wormwood)*,* a shrub from the Chinese medicinal plant [30]. This discovery led to YouYou Tu receiving the Nobel Prize in Medicine/Physiology in 2015. Currently, the Artemisinin-based combination therapies (ACTs) are recommended by the World Health Organization (WHO) for treatment of the deadliest malaria, that is caused by the bite of female anopheles mosquitos, especially *Plasmodium falciparum* parasite [31].

## 3. Exploring Botanicals as Bio-Compatible Therapeutics

There is a total of 21,000 plants listed by WHO which are extensively used for medicinal purposes throughout the world. In India, approximately 2500 species have been discovered, out of which 150 are used commercially on a fairly large scale by the biopharmaceutical companies as mainstream medicine. India is the largest producer of medicinal plants and owns the name “the botanical garden of the world” [32]. Traditional Medicine (TM) offers interesting possibilities to combat MDR [33]. Herbal products show a wide spectrum of biological activities and thus are efficiently harnessed for managing diseases [34]. Merging nutritional and therapeutic prospective may provide a powerful weapon for controlling an array of diseases [35]. Secondary metabolites are the result of secondary plant metabolism and occur as an intermediate or end products [36]. The structures of secondary metabolites have been optimized during evolution so they act as defence mechanisms by interfering with molecular targets within the cell in herbivores, microbes, and plants [37]. In addition, many secondary metabolites can affect cell signalling or protect against oxidative or UV stress [38]. Herbal antibiotics work against both gram-negative and gram-positive bacteria. Plant derived antibiotics act predominantly through the breakdown of the cell wall and cell membranes of microorganisms, which can lead to the release of cellular content, protein binding domain disruption, enzyme inactivation, and ultimately leading to cell death [39]. Natural product derived drugs are called ideal antibiotics [40]. Consequently, they may not only be effective by killing the microorganism, but also by affecting cellular events in the pathogenic process. Therefore, the bacteria, fungi, and viruses do not have the ability to develop resistance to botanicals. There are billions of species of plants, fungi, bacteria, and animals that produce a plethora of chemical compounds with equally diverse chemical structures, activities, and pharmacological properties, many of which are still unknown to humans. Hence, nature is the exclusive and ultimate source of all such drugs. From a drug discovery perspective, plant chemical molecules will hit the drug target at specific sites and rule over the synthetic compound. Thus, plant chemical constituents are one of the richest hot spots for most significant new drug discoveries [41]. Herbal medicines have gained special interest in recent years as a subject of both commercial and scientific interests [42].

## 4. Plant Secondary Metabolites: Key Target Player

Plants synthesize secondary metabolites (small organic molecules) that are not required for their normal growth or development but are essentially required for reproduction and defence mechanism against bacteria, fungus, virus, vertebrates, etc. These products have a great potential to act as drugs [43,44]. Many secondary metabolites are involved in the antagonistic relationship between plants and other organisms, but also in mutualistic ones (i.e., plants/pollinators, plants/disseminators, nitrogen-fixing plants/microorganisms, etc.) [45]. Secondary metabolites are the heterogeneous group of naturally occurring compounds, which have been used to treat various diseases [46]. The biochemistry of medicines based on traditional natural products have made a tremendous contribution to public healthcare and has boosted the development of affordable medicines globally [47]. Secondary metabolites have been investigated extensively since the 1850s [48]. Their classification can be based on the chemical composition (containing nitrogen or not), chemical structure (e.g., having rings, containing a sugar), the biosynthetic pathway (e.g., phenylpropanoid, which produces tannins) or their solubility. They are divided into three large categories, namely alkaloids, terpenes, and phenolics [48,49]. The greater part of plant derived compounds are phytochemicals, and secondary metabolites, which play a dominant role as antimicrobials and antivirals and are classified in many groups such as, alkaloids, phenolics, polyphenols, flavonoids, quinones, tannins, coumarins, terpenes, lectins and polypeptides, saponins, etc. [49,50,51]. Due to the high demand on the pharmaceutical and food industries, antimicrobial properties of polyphenols have been fuelled to develop new food preservatives to avoid synthetic preservatives and to develop novel therapies for the treatment of different microbial infections to combat microbial resistance against conventional antibiotics.

## 5. Mechanism of Action of Botanicals: Proof Based Research

Ethnobotany is located at the heart of natural science that deals with the relationship between plants and humans. Scientists explore the knowledge of ethnobotanical for the bioprocessing of new drugs together with new food crops to feed the growing human population. In order to identify new potential bioactive compounds from plant species, the knowledge of ethnobotany is essential. This emerging scientific field is accelerating the discovery of new biologically and chemically active natural compounds [52]. Table 2 gives details about classical medicines developed from different plant species [53]. Synthetic drugs and natural products differ significantly in terms of the frequency and configuration of different radicals [54]. The natural products have less nitrogen, sulphur, phosphorus, halogens, and exhibit overall enhanced scaffold variety, molecular complexity, stereo chemical abundance, diversity in the ring system, and carbohydrate contents [55]. Additionally, plant products have the ability to modify or inhibit protein–protein interactions, thus presenting themselves as effective modulators of immune response, mitosis, apoptosis, and signal transduction [56]. Bacteria are unable to develop resistance to multiple chemically complex phytochemicals present in plant extracts [57]. The use of traditional medicines clearly depicts how biologically potential active compounds can kill the pathogens and can stop further advance of the disease. During the early 19th century, new research began that involve the isolation and purification of plant active compounds with the help of different traditional techniques and methodologies. For example, there was a discovery of painkiller (analgesic) drugs codeine and morphine from the opium plant (*Papaver somniferum* L.), cocaine from *Erythroxylum coca* Lam., and quinine from *Cinchona calisaya* Wedd, etc., which are currently in use [58]. The microbial cell can be affected by secondary metabolites in several different ways. These include:

- Disruption of cell membrane functions and structure [59]
- Interference with intermediary metabolism [60]
- Interruption of DNA/RNA synthesis and function [61]
- Interruption of normal cell communication (quorum sensing) [62]
- Induction of coagulation of cytoplasmic constituents [63].

The plant-extracted product may exert its anti-microbial activity, not by killing the microorganism itself, but by affecting several key events in the pathogenic process [64,65]. The anti-diarrheal activity of extracts collected from the guava leaf is one such example. The extract of guava leaf is not bactericidal, but affects crucial pathogenic events of colonization and toxin production by diarrheal pathogens [66]. The study by Rajasekaran et al. [67] also demonstrates that the presence of multiple antiviral components in plant extracts interfaces with different viral proteins at various stages of viral replication. A study by Gupta et al. [68] showed that extracts from *Alpinia galanga* (L.) Willd. are effective against multi-drug resistant isolates of *M. tuberculosis*. The efficacy of extracts under aerobic and anaerobic conditions is suggestive of varied mode(s) of action by phytoactive components present in the plant extract. This innovation led to the discovery of different biologically active plant compounds. Aqueous extracts of the Chilean soapbark tree (*Quillaja saponaria* Molina) contain many physiologically active triterpenoid saponins [69]. These saponins have been tested for use in animal and human vaccines as they exhibit strong adjuvant activity [70]. Because of the strong immune-enhancing activity of *Quillaja* spp. extracts, it may lead to a reduction in virus infection in vivo and some researchers have suggested that *Quillaja* saponins prevent attachment of rotavirus by forming a ‘coat’ on the epithelium of the host’s small intestine [71]. Hence, ethnobotany is also called the “medicine of life” [42].

## 6. Herbal Drug Formulations

Undoubtedly, there are many herbal drug formulations (Table 3) commercially available in the market and regularly used by patients. An ethnopharmacologist selects desired plants from a variety of sources, and then a phytochemist extracts active compounds from the selected plants followed by biological identification and screening assays in order to identify the potential pharmacological activity. Next, the exact molecular mechanism, the mode of action, and therapeutic target will be studied by a molecular biologist, which is known as in vitro drug discovery. Following these studies, comes the in vivo tests to mainly check the efficacy, toxicity, and the effect and interaction of the new drug in a complete organism, preferably in mammals. The final identification from many compounds to a single potential active compound requires a multidisciplinary approach termed as pharmacognosy. A remarkably success story of an herbal product is the curcumin (natural polyphenols) isolated from *Curcuma longa* L. It is sold in the market in many forms such as herbal supplements, food additives to increase flavour and aroma, cosmetics ingredients, and as a colouring agent in many food items [72]. This yellow chemical is the most outstanding chemotherapeutic agent studied so far. The curcumin is used as medicine in both the ancient system of medicine TIM and TCM for the treatment of many diseases [73]. The potential power of curcumin to regulate several essential biological functions inside the body, such as redox status, protein kinases, transcription factors, adhesion molecules, and cytokines makes curcumin a key player in many different diseases like antineoplastic, anti-proliferative, anti-aging, anti-inflammatory, anti-angiogenic, scar formation, and anti-oxidant agent. Hence, it is used continuously to treat several diseases. This includes acquired immunodeficiency disease (AIDS), inflammatory bowel diseases, neurodegenerative diseases, inflammatory bowel diseases, cancer, cardiovascular diseases, allergies, rheumatoid arthritis, diabetes, psoriasis, scleroderma, asthma and bronchitis, and renal ischemia [74]. Another important example of a plant used in TCM is *Cannabis sativa* L., usually used to treat constipation, malaria, rheumatic pains as well as pain during childbirth. *C. sativa* L. is an important plant from the drug discovery point of view as it contains more than 60 terpenophenolic compounds called phytocannabinoids. For the last two centuries, *Cannabinoids* have been used as supportive drugs for those patients whose treatment requires radiation or chemotherapies [75].

## 7. Bio-Enhancers: Combining Traditional and Modern Medicine

Modern medicines have many disadvantages. A significant one is the side effects, which can impair the quality of life. Moreover, the treatment cost is also higher than can be afforded by millions of patients who are living in developing nations. Hence, there is no doubt that there is an unmet need for new effective and less harmful drugs, natural product derived compounds, and for promising new drugs that overcome the disadvantages of using modern medicines [82]. As increased acquired resistance to conventional antibiotics is evident, it is logical to attempt combining a therapy of standard antibiotics with plant extracts that possess bio-enhancing activity to attain bactericidal synergism [83,84]. Combination therapy can be used for:

- Expansion of antimicrobial spectrum
- Prevention of the emergence of drug resistant mutants
- Minimizing the toxicity level.

## 8. Bio-Enhancers May Act By

- Increasing drug ADME (Absorption, Distribution, Metabolism, and Excretion)
- Modulating biotransformation of drugs in the liver and intestines
- Modulating active transport phenomenon
- Decreasing elimination
- Boost the immune system.

It is often believed that bacteria cannot develop resistance to botanicals [85]. It is possible that bacteria may develop resistance to herbal treatment if only one active principal with a specific target is involved [86], a situation similar to an antibiotic. Nevertheless, since the available literature on bacteria developing resistance to botanicals is limited then further research is required on mechanisms to study the development of this resistance [87].

## 9. Concluding Remarks and Future Prospects

In the current scenario, the problem of emerging MDR bacteria is posing a global medical threat and is continuously challenging the scientific community. Understanding the key molecular mechanisms involved in the screening of bioactive small molecule compounds has become a major challenge for drug discovery scientists. The reduction of efficacy and the increase of toxicity of synthetic drugs is further aggravating the problem. This has led researchers to look towards herbal drugs for a solution, as they are now known to play a crucial role in the development of effective therapeutics. The success story of artemisinin serves as the best example for encouraging ethnobotanists to pursue research on more plant derived drugs to combat MDR [88]. Biotechnology is the most powerful tool and will spark the manufacture of new drugs from plant sources at a much faster and controlled process [89]. It boosts the pipeline of drug discovery and development [90,91,92].

**Figure 1:**
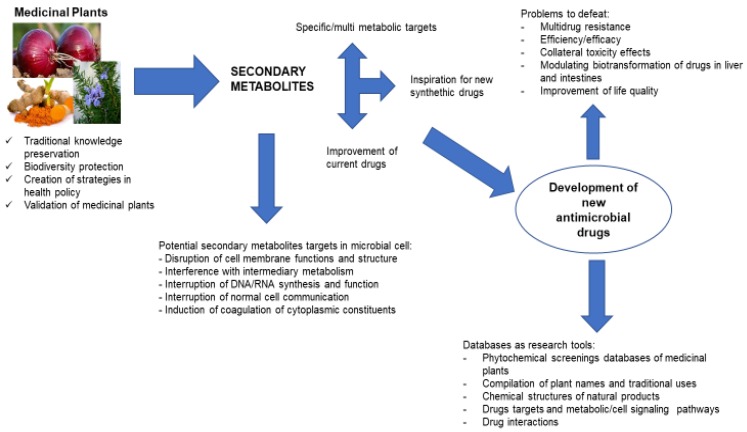
Flow diagram summarizing the potential of natural products in the discovery of new antimicrobial agents.

In conclusion, there is an urgent need to continue research models to support the development of botanicals to counter drug resistant microbes, as well as regulatory reforms of clinical development programs. The use of botanical medicines is accelerating and improving the channel of drug development. There are several reasons to use herbal medicines of which two may play pivotal roles. First, herbal treatment provides other mechanisms of action, encompassing in many cases a single drug to treat a single disease. Second, the utilization of unique traditional knowledge of herbal medicine has great potential to generate biocompatible, cost effective solutions and will hasten the discovery of new medicines (Figure 1).

Indeed, it is an important call for coordination and collaboration between the World Health Organization (WHO), the Food and Drug Administration (FDA), European Medicines Agency (EMA), the biotech companies, pharmaceutical industry, and several other regulatory agencies globally to provide clear guidelines for the discovery and development of herbal drugs to utilize the vast potential of traditional medicine for development of drugs for different diseases. Undoubtedly, medicinal phytochemicals are important natural resources for future drug discoveries, and only a small percentage of the phytochemical properties of medicinal plants have been investigated. Full effort must be given to explore and evaluate potential molecular characterization of the medicinal compounds with the help of databases and interdisciplinary group efforts. In the end, the finding of more effective and less toxic drugs will benefit the global population.

## Figures and Tables

**Table 1:** Databases available for natural products screening.

Database Name	Hyperlink	Purpose of the Database
Indian Medicinal Plants, Phytochemistry and Therapeutics (IMPPAT)	http://cb.imsc.res.in/imppat/home	The database contains the largest phytochemicals list of the Indian medicinal plants
METLIN Metabolomics	https://metlin.scripps.edu/landing_page.php?pgcontent=mainPage	MS/MS metabolite database
Cardiovascular Disease Herbal Database (CVDHD)	http://pkuxxj.pku.edu.cn/CVDHD	Database specialized in herbs used for cardiovascular diseases for drug discovery
KNApSAcK	http://www.knapsackfamily.com/KNApSAcK/	Database to find species-metabolite relationship
Dr. Duke’s Phytochemical & Ethnobotanical Database	https://phytochem.nal.usda.gov/phytochem/search/list	Database for searching chemical, bioactivity, and ethnobotany information
Traditional Chinese Medicine Integrated Database (TCMID)	http://119.3.41.228:8000/	A complete database of the TCM, including formulae, herbs, and herbal ingredients
TCM@Taiwan	http://tcm.cmu.edu.tw/	The world’s largest database of TCM for drug screening in silico
TCM-Mesh	http://mesh.tcm.microbioinformatics.org	Database for network pharmacology analysis of TCM preparations
DrugBank	https://www.drugbank.ca/	Bioinformatics and cheminformatics database for drug data and drug target information
Search Tool for Interacting Chemicals (STITCH)	http://stitch.embl.de/	Database specialized in the known and predicted interactions between chemicals and proteins
Medicinal Plant Genomics Resource (MPGR)	http://medicinalplantgenomics.msu.edu	Website specialized in the genome and metabolome of medicinal plants
PubChem	https://pubchem.ncbi.nlm.nih.gov/	Chemistry database
Therapeutic Targets Database (TTD)	https://db.idrblab.org/ttd/	Database of therapeutic proteins and nucleic acid targets, targeted disease, pathway information and drugs used for the targets
NuBBE Database	http://nubbe.iq.unesp.br/portal/nubbe-search.html	Database is helpful for studies on naturally occurring bioactive compounds, molecular and physicochemical properties
SistematX	https://sistematx.ufpb.br/	An online Cheminformatics tool for secondary metabolites data management
Super Natural II	http://bioinformatics.charite.de/supernatural	Natural products database which contains ∼325,508 natural compounds (NCs)/ molecules
InterBioScreen Natural Products Database	https://www.ibscreen.com/natural-compounds	The database contains over 68 000 highly diverse natural compounds

**Table 2:** Examples of successful drugs derived from medicinal plants.

Plant Derived Drugs/Molecules	Plant Species
Aspirin	*Filipendula ulmaria* (L.) Maxim
Codeine	*Papaver somniferum* L.
Papaverine	*Papaver somniferum* L.
Colchicine	*Colchicum autumnale* L.
Digoxin and digitoxin	*Digitalis purpurea* L.
Cannabidiol	*Cannabis sativa* L.
Tetrahydrocannabinol	*Cannabis sativa* L.
Vinblastine and vincristine	*Catharanthus roseus* (L.) G. Don
Artemisinin	*Artemisia annua* L.
Galantamine (Reminyl^®^)	*Galanthus woronowii* Losinsk.
Apomorphine hydrochloride (Apokyn^®^)	*Papaver somniferum* L.
Tiotropium bromide (Spiriva^®^)	*Atropa belladonna* L.
Paclitaxel (Taxol^®^)	*Taxus brevifolia* Nutt.
Vinblastine and vincristine	*Catharanthus roseus* (L.) G. Don
Paclitaxel	*Taxus brevifolia* Nutt. *& Taxus chinensis* (Pilg.) Rehder
Camptothecin	*Camptotheca acuminate* Decne.
Allicin (diallylthiosulfnate)	*garlic* (*Allium sativum* L.)

**Table 3:** Examples of herbal molecules used in the industry of health and food.

Functional Properties	Plant Molecules	References
Food and Nutrition	Vitamins, flavonols carotenoids, anthocyanins catechins, lycopene, genistein, daidzein, resveratrol, plant-based/non-dairy milk	Rahal et al., 2014 [76]
Health	Taxol, quinine, artemisinin, morphine, minerals, polysaccharides, amino acids, enzymes, vitamins,	Fridlender et al., 2015 [77] Habeeb et al., 2007 [78]
Sweeteners	Stevioside, rebaudioside A (C_44_H_70_O_230)_	Soejarto et al., 2019 [79]
Aroma/flavours	Menthol, benzyl acetate, vanillin, 2-phenylethel alcohol, eugenol, limonene, linalool, ionones, anethole, cinnamaldehyde	Schwab et al., 2008 [80] Altemimi et al.,2017 [81]

## References

[1] O’Neill J. (2016). Tackling Drug-Resistant Infections Globally: Final Report and Recommendations–The Review on Antimicrobial Resistance Chaired by Jim O’Neill.

[2] De Kraker M.E.A., Stewardson J., Harbath S. (2016). Will 10 million people die a year due to antimicrobial resistance by 2050?. PLoS Med..

[3] Brown E.D., Wright G.D. (2016). Antibacterial drug discovery in the resistance era. Nature.

[4] Chandra H., Bishnoi P., Yadav A., Patni B., Mishra A.P., Nautiyal A.R. (2017). Antimicrobial Resistance and the Alternative Resources with Special Emphasis on Plant-Based Antimicrobials—A Review. Plants.

[5] Chen S., Song J., Sun C., Xu J., Zhu Y., Verpoorte R., Fan T.P. (2015). Herbal genomics: Examining the biology of traditional medicines. Science.

[6] Newman D.J., Cragg G.M. (2016). Natural Products as Sources of New Drugs from 1981 to 2014. J. Nat. Prod..

[7] Chin Y.W., Balunas M.J., Chai H.B., Kinghorn A.D. (2006). Drug discovery from natural sources. AAPS J..

[8] Robinson M.M., Zhang X. (2011). The World Medicines Situation 2011, Traditional Medicines: Global Situation, Issues and Challenges.

[9] Chavan S.S., Damale M.G., Devanand B. (2018). Antibacterial and Antifungal Drugs from Natural Source: A Review of Clinical Development.

[10] Tortorella E., Tedesco P., Esposito P.F., January G.G., Fani R., Jaspars M., de Pascale D. (2018). Antibiotics from deep-sea microorganisms: Current discoveries and perspectives. Mar. Drugs.

[11] Penesyan A., Khelleberg S., Egan S. (2010). Development of novel drugs from marine surface associated microorganisms. Mar. Drugs.

[12] David B., Wolfender J.-L., Dias D.A. (2015). The pharmaceutical industry and natural products: Historical status and new trends. Phytochem Rev..

[13] Pye C.R., Bertin M.J., Lokey R.S., Gerwick W.H., Linington R.G. (2017). Retrospective analysis of natural products provides insights for future discovery trends. Proc. Natl. Acad. Sci. USA.

[14] Boucher H.W., Ambrose P.G., Chambers H.F., Ebright R.H., Jezek A., Murray B.E., Newland J.G., Ostrowsky B., Rex J.H. (2017). White paper: Developing antimicrobial drugs for resistant pathogens, narrow-spectrum indications, and unmet needs. J. Infect. Dis..

[15] Luepke K.H., Suda K.J., Boucher H., Russo R.L., Bonney M.W., Hunt T.D., Mohr J.F. (2017). Past, present, and future of antibacterial economics: Increasing bacterial resistance, limited antibiotic pipeline, and societal implications. Pharmacotherapy.

[16] Rodrigues T., Reker D., Schneider P., Schneider G. (2016). Counting on natural products for drug design. Nat. Chem..

[17] Patwardhan B., Warude D., Pushpangadan P., Bhatt N. (2005). Ayurveda and traditional Chinese medicine: A comparative overview. Evid. Based Complement. Altern. Med..

[18] Jianjun T., Zhou Z. (2016). Traditional Chinese Medicine as Prevention and Treatment Strategies of HIV Infection. J. Drug.

[19] Butler M.S., Robertson A.A.V., Cooper M.A. (2014). Natural product and natural product derived drugs in clinical trials. Nat. Prod. Rep..

[20] Wood M. (2017). The Book of Herbal Wisdom: Using Plants as Medicines.

[21] Blair J.M.A., Webber M.A., Baylay A.J., Ogbolu D.O., Piddock L.J. (2015). Molecular mechanisms of antibiotic resistance. Nat. Rev. Microbiol..

[22] Atanasov A.G., Waltenberger B., Pferschy-Wenzig E.M., Linder T., Wawrosch C., Uhrin P., Temml V., Wang L., Schwaiger S., Heiss E.H. (2015). Discovery and resupply of pharmacologically active plant-derived natural products: A review. Biotechnol. Adv..

[23] National Center for Complementary and Integrative Health (2017). Natural Product Libraries.

[24] Owen J.G., Reddy B.V., Ternei M.A., Charlop-Powers Z., Calle P.Y., Kim J.H., Brady S.F. (2013). Mapping gene clusters within arrayed metagenomic libraries to expand the structural diversity of biomedically relevant natural products. Proc. Natl. Acad. Sci. USA.

[25] Medina-Franco J.L. (2015). Discovery and development of lead compounds from natural sources using computational approaches. Evidence-Based Validation of Herbal Medicine.

[26] Baxevanis A.D., Bateman A. (2015). The importance of biological databases in biological discovery. Curr. Protoc. Bioinform..

[27] Prachayasittikul V., Worachartcheewan A., Shoombuatong W., Songtawee N., Simeon S., Prachayasittikul V., Nantasenamat C. (2015). Computer-aided drug design of bioactive natural products. Curr. Top. Med. Chem..

[28] Lagunin A.A., Goel R.K., Gawande D.Y., Pahwa P., Gloriozova T.A., Dmitriev A.V., Ivanov S.M., Rudik A.V., Konova V.I., Pogodin P.V. (2014). Chemo-and bioinformatics resources for in silico drug discovery from medicinal plants beyond their traditional use: A critical review. Nat. Prod. Rep..

[29] Xin-Zhuan S., Miller L.H. (2015). The discovery of artemisinin and the Nobel Prize in Physiology or Medicine. Sci. China Life Sci..

[30] Shen Q., Zhang L., Liao Z., Brodelius P.E. (2018). The Genome of *Artemisia annua* Provides Insight into the Evolution of Asteraceae Family and Artemisinin Biosynthesis. Mol. Plant.

[31] Sen S., Chakraborty R. (2017). Revival, modernization and integration of Indian traditional herbal medicine in clinical practice: Importance, challenges and future. J. Tradit. Complement. Med..

[32] Yuan H., Ma Q., Ye L., Piao G. (2016). The traditional medicine and modern medicine from natural products. Molecules.

[33] Gupta P.D., Birdi T.J. (2017). Development of botanicals to combat antibiotic resistance. J. Ayurveda Integr. Med..

[34] Shils M.E., Shike M. (2006). Modern Nutrition in Health and Disease.

[35] Chen M.C., Hao Z., Tian Y., Zhang Q.Y., Gao P.J., Jin J.L. (2013). Different effects of six antibiotics and ten traditional Chinese medicines on shiga toxin expression by Escherichia coli O157:H7. Evid. Based Complement. Altern. Med..

[36] Stefanovic O. (2012). Comic Synergistic antibacterial interaction between *Melissa officinalis* extracts and antibiotics. J. Appl. Pharm. Sci..

[37] Kasote D.M., Katyate S.S., Hedge M.V., Bae H. (2015). Significance of antioxidant potential of plants and its relevance therapeutic applications. Int. J. Biol. Sci..

[38] Fair R.J., Tor Y. (2014). Antibiotics and bacterial resistance in the 21st century. Perspect. Med. Chem..

[39] Singh S.B., Young K., Silver L.L. (2017). What is an “ideal” antibiotic? Discovery challenges and path forward. Biochem Pharmacol..

[40] Alvin A., Miller K.I., Neilan B.A. (2014). Exploring the potential of endophytes from medicinal plants as sources of antimycobacterial compounds. Microbiol. Res..

[41] Leonti M. (2013). Traditional medicines and globalization: Current and future perspectives in ethnopharmacology. Front. Pharmacol..

[42] Wink M. (2015). Modes of action of herbal medicines and plant secondary metabolites. Medicines.

[43] Boy H.I., Rutilla A.J., Santos K.A., Ty A.M., Alicia I.Y., Mahboob T., Tangpoong J., Nissapatorn V. (2018). Recommended Medicinal Plants as Source of Natural Products: A Review. Digit. Chin. Med..

[44] Mawalagedera S.M., Symonds M.R., Callahan D.L., Gaskett A.C., Rønsted N. (2019). Combining evolutionary inference and metabolomics to identify plants with medicinal potential. Front. Ecol. Evol..

[45] Leicach S.R., Chludil H.D. (2014). Plant secondary metabolites: Structure–activity relationships in human health prevention and treatment of common diseases. Stud. Nat. Prod. Chem..

[46] Moloney M.G. (2016). Natural products as a source for novel antibiotics. Trends Pharmacol. Sci..

[47] Soukup R.W., Soukup K. (2015). The Series Progress in the Chemistry of Organic Natural Products: 75 Years of Service in the Development of Natural Product Chemistry. Progress in the Chemistry of Organic Natural Products 100.

[48] Kabera J.N., Semana E., Mussa A.R., He X. (2014). Plant secondary metabolites: Biosynthesis, classification, function and pharmacological properties. J. Pharm. Pharmacol..

[49] Alamgir A.N.M. (2017). Pharmacognostical Botany: Classification of Medicinal and Aromatic Plants (MAPs), Botanical Taxonomy, Morphology, and Anatomy of Drug Plants. Therapeutic Use of Medicinal Plants and Their Extracts.

[50] O’Connor S.E. (2015). Engineering of secondary metabolism. Ann. Rev. Genet..

[51] Katz L., Baltz R.H. (2016). Natural product discovery: Past, present, and future. J. Ind. Microbiol. Biot..

[52] Garnatje T., Peñuelas J., Vallès J. (2017). Ethnobotany, phylogeny, and omics for human health and food security. Trends Plant Sci..

[53] Hussain M.S., Fareed S., Ansari S., Rahman M.A., Ahmad I.Z., Saeed M. (2012). Current approaches toward production of secondary plant metabolites. J. Pharm. Bioallied Sci..

[54] Szychowski J., Truchon J.-F., Bennani Y.L. (2014). Natural products in medicine: Transformational outcome of synthetic chemistry. J. Med. Chem..

[55] Jakobek L. (2015). Interactions of polyphenols with carbohydrates, lipids and proteins. Food Chem..

[56] Vadhana P., Singh B.R., Bharadwaj M., Singh S.V. (2015). Emergence of herbal antimicrobial drug resistance in clinical bacterial isolates. Pharm. Anal. Acta..

[57] Ody P. (2017). The Complete Medicinal Herbal: A Practical Guide to the Healing Properties of Herbs.

[58] De Filippis L.F., Mohamed M.A., Ahmad P. (2016). Plant secondary metabolites: From molecular biology to health products. Plant-Environment Interaction: Responses and Approaches to Mitigate Stress.

[59] Chitemerere T.A., Mukanganyama S. (2014). Evaluation of cell membrane integrity as a potential antimicrobial target for plant products. BMC Complement. Altern. Med..

[60] Anandhi D., Srinivasan P.T., Kumar G., Jagatheesh S. (2014). DNA fragmentation induced by the glycosides and flavonoids from *C. coriaria*. Int. J. Curr. Microbiol. Appl. Sci..

[61] Zhao X., Zhao F., Zhong N. (2018). Quorum Sensing Inhibition and Anti-Biofilm Activity of Traditional Chinese Medicines. Food Safety-Some Global Trends.

[62] Radulovic N.S., Blagojevic P.D., Stojanovic-Radic Z.Z., Stojanovic N.M. (2013). Antimicrobial plant metabolites: Structural diversity and mechanism of action. Curr. Med. Chem..

[63] Mogosanu G.D., Grumezescu A.M., Huang K.S., Bejenaru L.E., Bejenaru C. (2015). Prevention of microbial communities: Novel approaches based natural products. Curr. Pharm. Biotechnol..

[64] Bazaka K., Jacob M.V., Chrzanowski W., Ostrikov K. (2015). Anti-bacterial surfaces: Natural agents, mechanisms of action, and plasma surface modification. RSC Adv..

[65] Díaz-de-Cerio E., Verardo V., Gómez-Caravaca A.M., Fernández-Gutiérrez A., Segura-Carretero A. (2017). Health effects of *Psidium guajava* L. leaves: An overview of the last decade. Int. J. Mol. Sci..

[66] Rajasekaran E., Palombo E., Yeo T., Ley D.L.S., Tu C.L., Malherbe F., Grollo L. (2014). Evidence of synergistic activity of medicinal plant extracts against neuraminidase inhibitor resistant strains of influenza viruses. Adv. Microbiol..

[67] Gupta P.D., Bhatter P.D., D’souza D., Tolani M., Daswani P., Tetali P. (2014). Evaluating the anti-*Mycobacterium tuberculosis* activity of *Alpinia galanga* (L.) Willd. Axenically under reducing oxygen conditions and intracellular assays. BMC Complement. Altern. Med..

[68] Ganjhu R.K., Mudgal P.P., Maity H., Dowarha D., Devadiga S., Nag S., Arunkumar G. (2015). Herbal plants and plant preparations as remedial approach for viral diseases. Virus Dis..

[69] Turmagambetova A.S., Alexyuk P.G., Bogoyavlenskiy A.P., Zaitseva I.A., Omirtaeva E.S., Alexyuk M.S., Sokolova N.S., Berezin V.E. (2017). Adjuvant activity of saponins from Kazakhstani plants on the immune responses to subunit influenza vaccine. Arch. Virol..

[70] Chanda S., Rakholiya K., Mendez-Vilas A. (2011). Indian combination therapy: Synergism between natural plant extracts and antibiotics against infectious diseases. Science Against Microbial Pathogens: Communicating Current Research and Technological Advances.

[71] Pan S.Y., Litscher G., Gao S.-H., Zhou S.-F., Yu Z.-L., Chen H.-Q., Zhang S.-F., Tang M.-K., Sun J.-N., Ko K.-M. (2014). Historical perspective of traditional indigenous medical practices: The current renaissance and conservation of herbal resources. Evid. Based Complement. Altern. Med..

[72] Nelson K.M., Dahlin J.L., Bisson J., Graham J., Pauli G.F., Walters M.A. (2017). The essential medicinal chemistry of curcumin: Miniperspective. J. Med. Chem..

[73] Chauhan M., Suman S., Amit R. (2014). Curcumin: A review. J. Appl. Pharm. Res..

[74] Aggarwal B.B., Kuzhuvelil B.H. (2009). Potential therapeutic effects of curcumin, the anti-inflammatory agent, against neurodegenerative, cardiovascular, pulmonary, metabolic, autoimmune and neoplastic diseases. Int. J. Biochem. Cell Biol..

[75] Le Boisselier R., Alexandre J., Lelong-Boulouard V., Debruyne D. (2017). Focus on cannabinoids and synthetic cannabinoids. Clin. Pharmacol. Ther..

[76] Rahal A., Verma A.K., Mahima A.K. (2014). Phytonutrients and nutraceuticals in vegetables and their multi-dimensional medicinal and health benefits for humans and their companion animals: A review. J. Biol. Sci..

[77] Fridlender M., Yoram K., Hinanit K. (2015). Plant derived substances with anti-cancer activity: From folklore to practice. Front. Plant Sci..

[78] Habeeb F., Shakir E., Bradbury F., Cameron P., Taravati M.R., Drummond A.J., Gray A.I., Ferro V.A. (2007). Screening methods used to determine the anti-microbial properties of Aloe vera inner gel. Methods.

[79] Soejarto D.D., Addo E.M., Kinghorn A.D. (2019). Highly sweet compounds of plant origin: From ethnobotanical observations to wide utilization. J. Ethnopharmacol..

[80] Schwab W., Davidovich-Rikanati R., Lewinsohn E. (2008). Biosynthesis of plant-derived flavour compounds. Plant J..

[81] Altemimi A., Lakhssassi N., Baharlouei A., Watson D., Lightfoot D. (2017). Phytochemicals: Extraction, isolation, and identification of bioactive compounds from plant extracts. Plants.

[82] Appendino G., Minassi A., Taglialatela-Scafati O. (2014). Recreational drug discovery: Natural products as lead structures for the synthesis of smart drugs. Nat. Prod. Rep..

[83] Dudhatra G., Mody S., Awale M., Patel H.B., Modi C.M., Kumar A., Kamani D.R., Chauhan B.N. (2012). A comprehensive review on pharmacotherapeutics of herbal bioenhancers. Sci. World. J..

[84] Tatiraju D., Bagade V., Karambelkar P., Jadhav V., Kadam V. (2013). Natural bioenhancers: An overview. J. Pharmacogn. Phytochem..

[85] Ruddaraju L.K., Pammi S.V.N., Guntuku G.S., Padavala V.W., Kolapalli V.R.M. (2019). A review on anti-bacterials to combat resistance: From ancient era of plants and metals to present and future perspectives of green nano technological combinations. Asian J. Pharm. Sci..

[86] Reker D., Rodrigues T., Perna A.M., Schenider P. (2014). Revealing the macromolecular targets of complex natural products. Nat. Chem..

[87] Almabruk K.H., Dinh L.K., Philmus B. (2018). Self-resistance of natural product producers: Past, present and future focusing on self-resistant protein variants. ACS Chem. Biol..

[88] Tu Y. (2016). Artemisinin—A gift from traditional Chinese medicine to the world (Nobel lecture). Angew. Chem. Int. Ed..

[89] Alamgir A.N.M. (2018). Biotechnology, In Vitro Production of Natural Bioactive Compounds, Herbal Preparation, and Disease Management (Treatment and Prevention). Therapeutic Use of Medicinal Plants and Their Extracts.

[90] Gandhi S.G., Vidushi M., Yashbir S.B. (2015). Changing trends in biotechnology of secondary metabolism in medicinal and aromatic plants. Planta.

[91] Uhlenbrock L., Tegtmeier M., Sixt M., Schulz H. (2018). Natural Products Extraction of the Future—Sustainable Manufacturing Solutions for Societal Needs. Processes.

[92] Chakraborty P. (2018). Herbal genomics as tools for dissecting new metabolic pathways of unexplored medicinal plants and drug discovery. Biochimie.

